# Supplementary material for: Detecting sedimentation impacts to coral reefs resulting from dredging the Port of Miami, Florida USA
Source: PeerJ. 2016 Nov 17;4:e2711. doi: 10.7717/peerj.2711 (PMC5119242; doi:10.7717/peerj.2711)
Supplement: Table S1 — Percentage of scleractinian corals at each site with recent partial mortality, sediment accumulation, or sediment halo and number of corals from the belt transects partitioned by habitat type. Percentage of survey points with sediment over hardbottom and deep sediment from line-intercept transects. [file peerj-04-2711-s002.pdf]

Supplemental Table 1. Percentage of scleractinian corals at each Inner Reef north site with recent partial mortality, sediment accumulation, or sediment halo and number of corals from the belt transects partitioned by habitat type. Percentage of survey points with sediment over hardbottom and deep sediment from line-intercept transects.

Inner Reef north site	Number of Scleractinian Corals	Percent Corals with recent Partial Mortality	Percent Corals with Sediment Accumulation	Percent Corals with Sediment Halo	Percent Survey Points Characterized Sediment over Hardbottom	Percent Survey Points Characterized Deep Sediment over Hardbottom
100-RR	191	22%	20%	10%	52%	11%
100-LR	81	55%	64%	21%	61%	19%
200-RR	140	51%	48%	16%	63%	0%
200-LR	93	32%	37%	26%	81%	43%
300-RR	135	37%	22%	14%	40%	0%
300-LR	76	27%	25%	13%	45%	0%
500-RR	73	28%	19%	3%	27%	0%
500-LR	76	22%	26%	13%	43%	3%
700-LR	82	40%	15%	11%	53%	3%
Reference-RR	135	4%	4%	1%	3%	0%
Reference-LR	156	11%	3%	1%	1%	0%
