# Supplementary material for: Detecting sedimentation impacts to coral reefs resulting from dredging the Port of Miami, Florida USA
Source: PeerJ. 2016 Nov 17;4:e2711. doi: 10.7717/peerj.2711 (PMC5119242; doi:10.7717/peerj.2711)

Suppl Fig 1: Estimated reconstructed timeline of dredging, monitoring, and other coral reef disturbance events over approximately 112 weeks at the Port of Miami between late October 2014 and mid-December 2015. Black bars represent 10-week intervals.

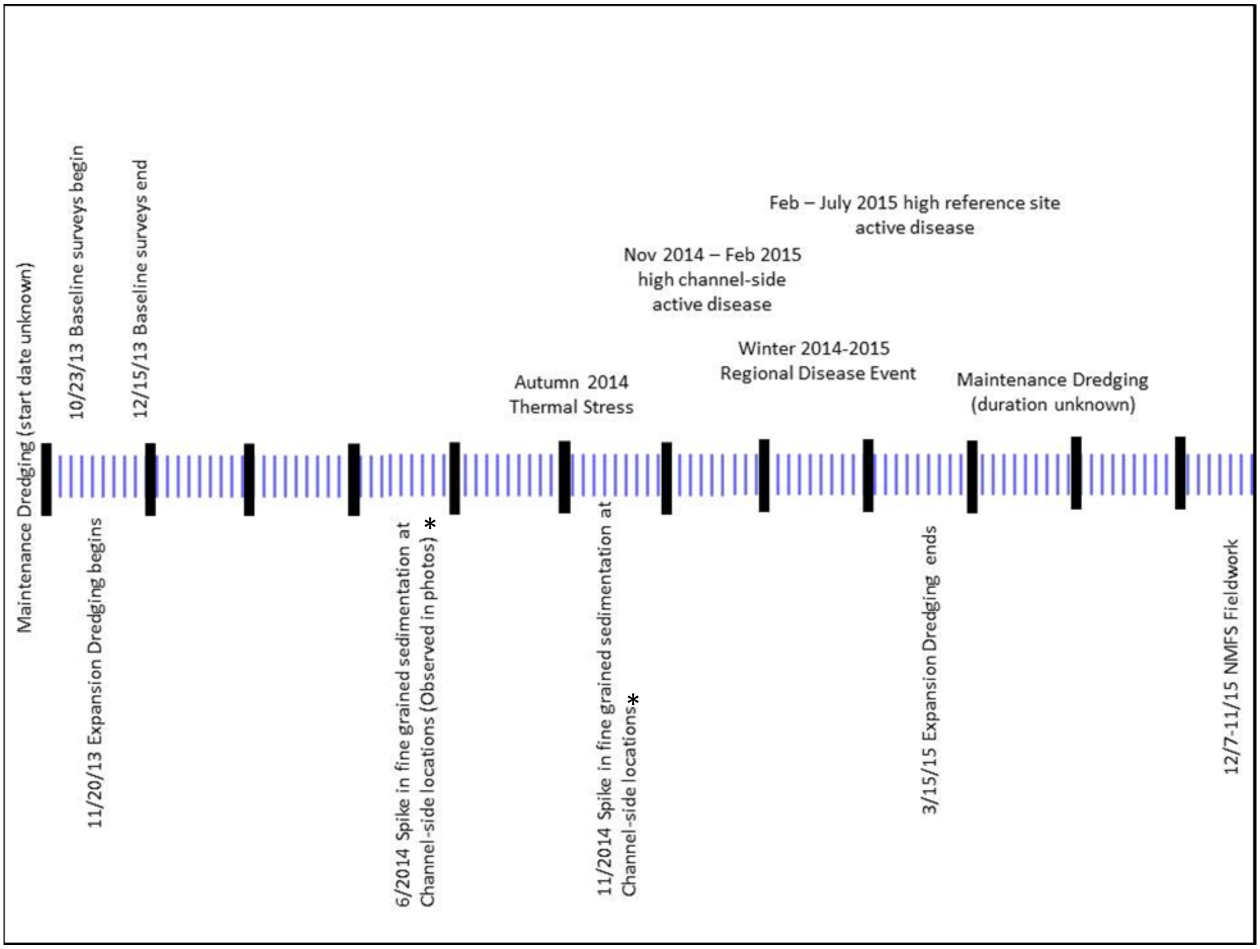

*Sedimentation data from USACE (2015b)

Suppl. Fig 2: All photos from the location surveyed 200 m from the channel in the Inner Reef north, Linear Reef habitat. A-B) Illustration of 'halo' pattern of partial mortality originating from partial burial of the colony with sediment (B taken after diver had manually removed sediment by fanning). C-D) Additional examples of edge burial (C) and 'halo' pattern of partial mortality showing sediment interaction (D). E-F) Partially buried soft corals in an area of 'deep sediment over hardbottom' (DSOHB).

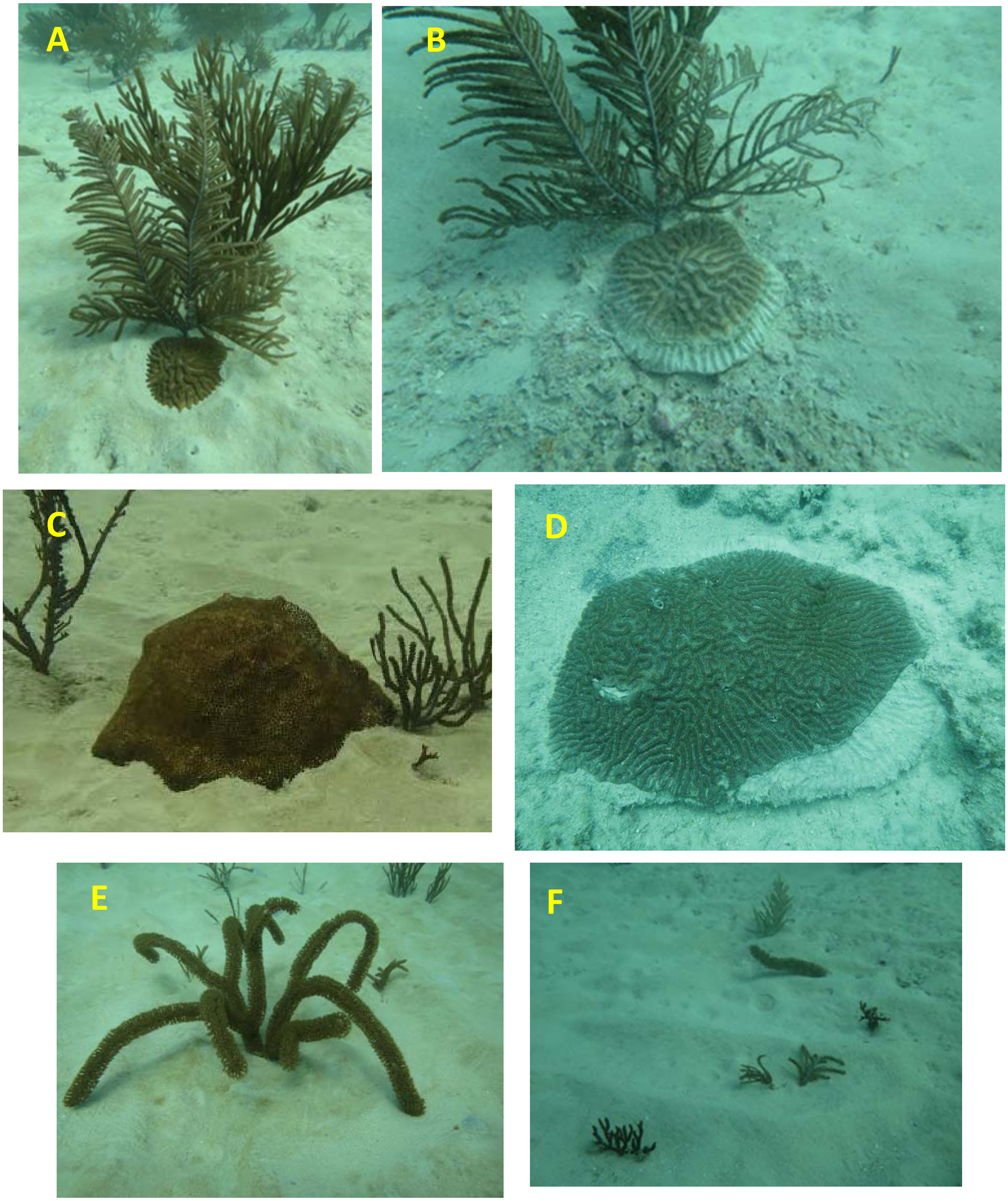

Suppl Fig 3A: Abbreviated time series photos illustrating common conditions. Channel-side Siderastrea siderea (R2N2 T3 C6) which was completely buried in sediment, apparently for several months, resulting in complete colony mortality

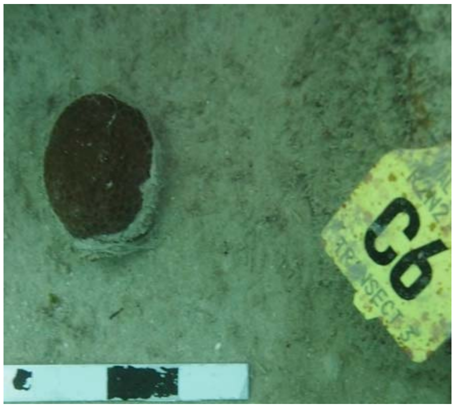

11/20/13

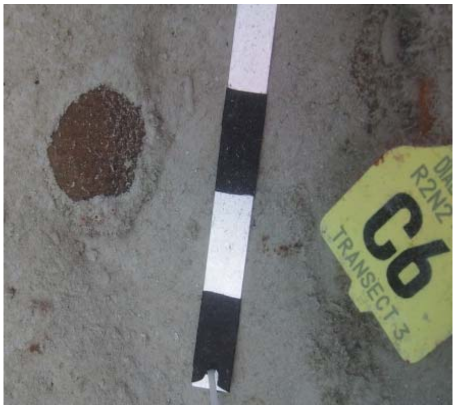

4/15/14

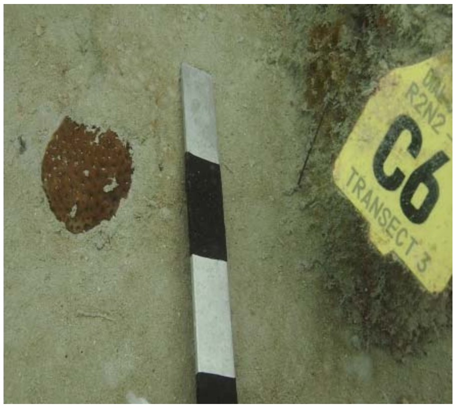

4/27/14

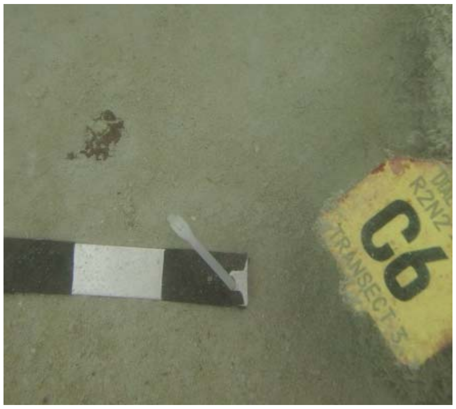

6/10/14

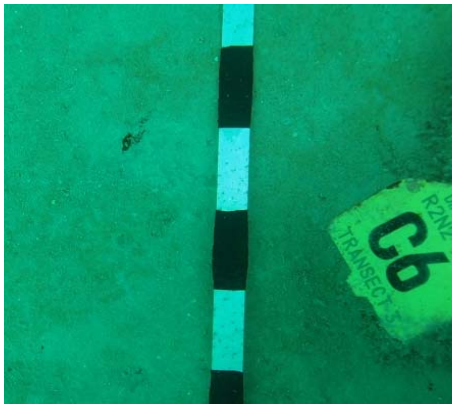

6/14/14

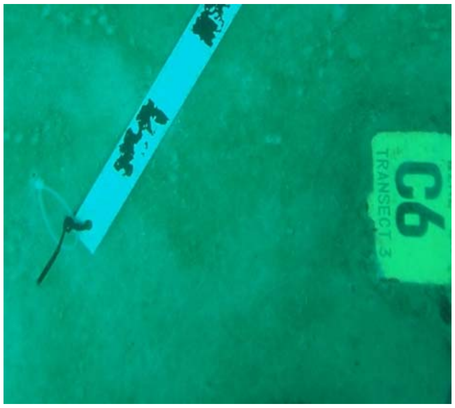

11/18/14

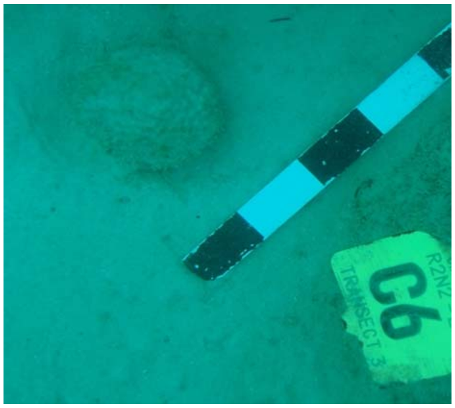

2/24/15

Suppl Fig 3B: Abbreviated time series photos illustrating common conditions. Channel-side *Meandrina meandrites* (R2N1 T2 C4) showing substantial partial burial in sediment. Disease signs are apparent both in direct combination with heavy sedimentation (June-July 2014, prior to the onset of warm thermal stress) and more severely later on (fall-winter 2014). Complete colony mortality attributed to disease.

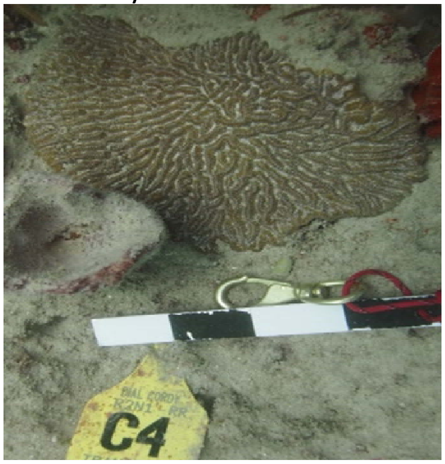

11/12/13

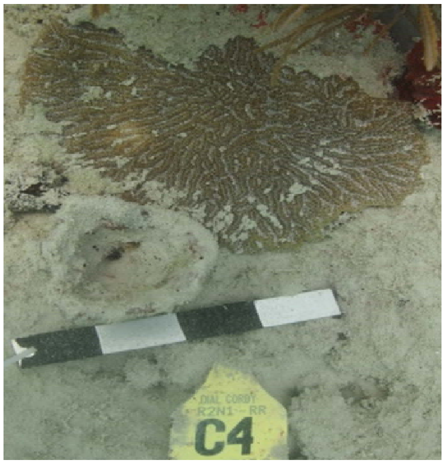

4/25/14

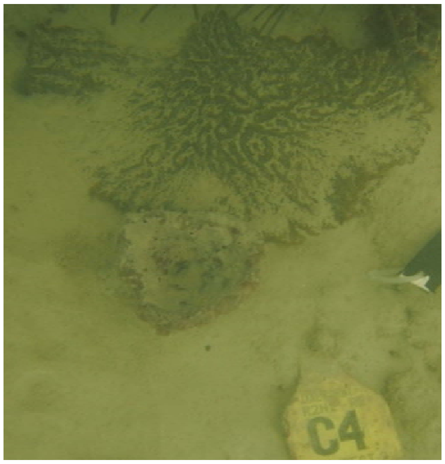

6/16/14

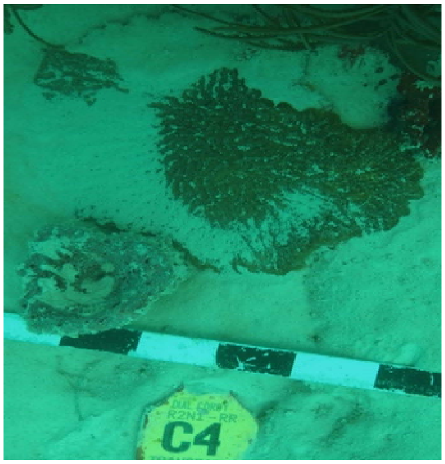

6/22/14

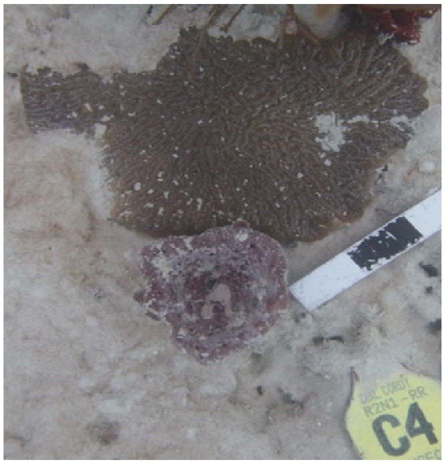

7/9/14

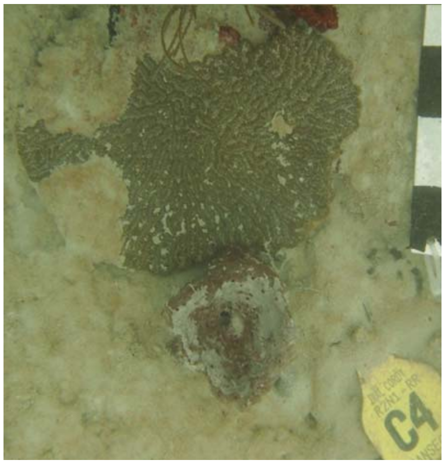

8/8/14

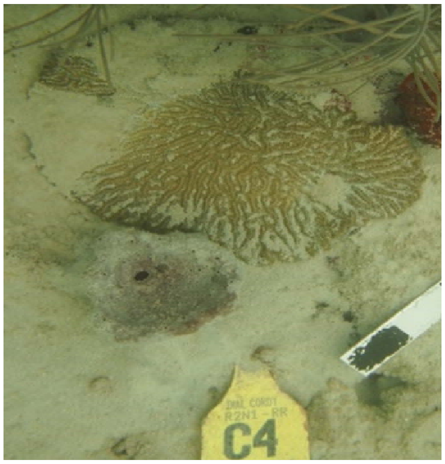

9/19/14

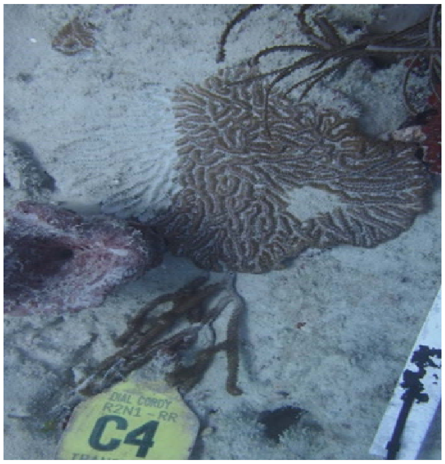

11/13/14

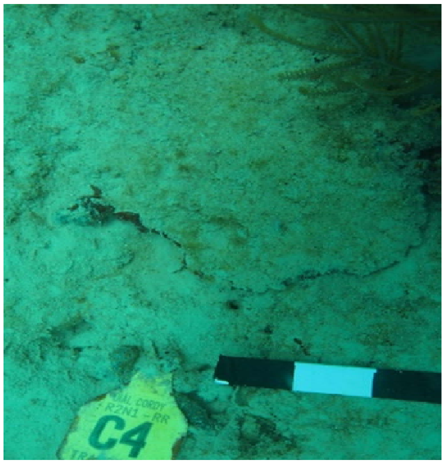

7/6/15

Suppl Fig 3C: Abbreviated time series photos illustrating common conditions. Reference colony of *Siderastrea siderea* (R2NC1 T1 C7) showing some apparent interaction with sediment along the colony margin throughout. This colony bleached in fall 2014 and subsequently recovered.

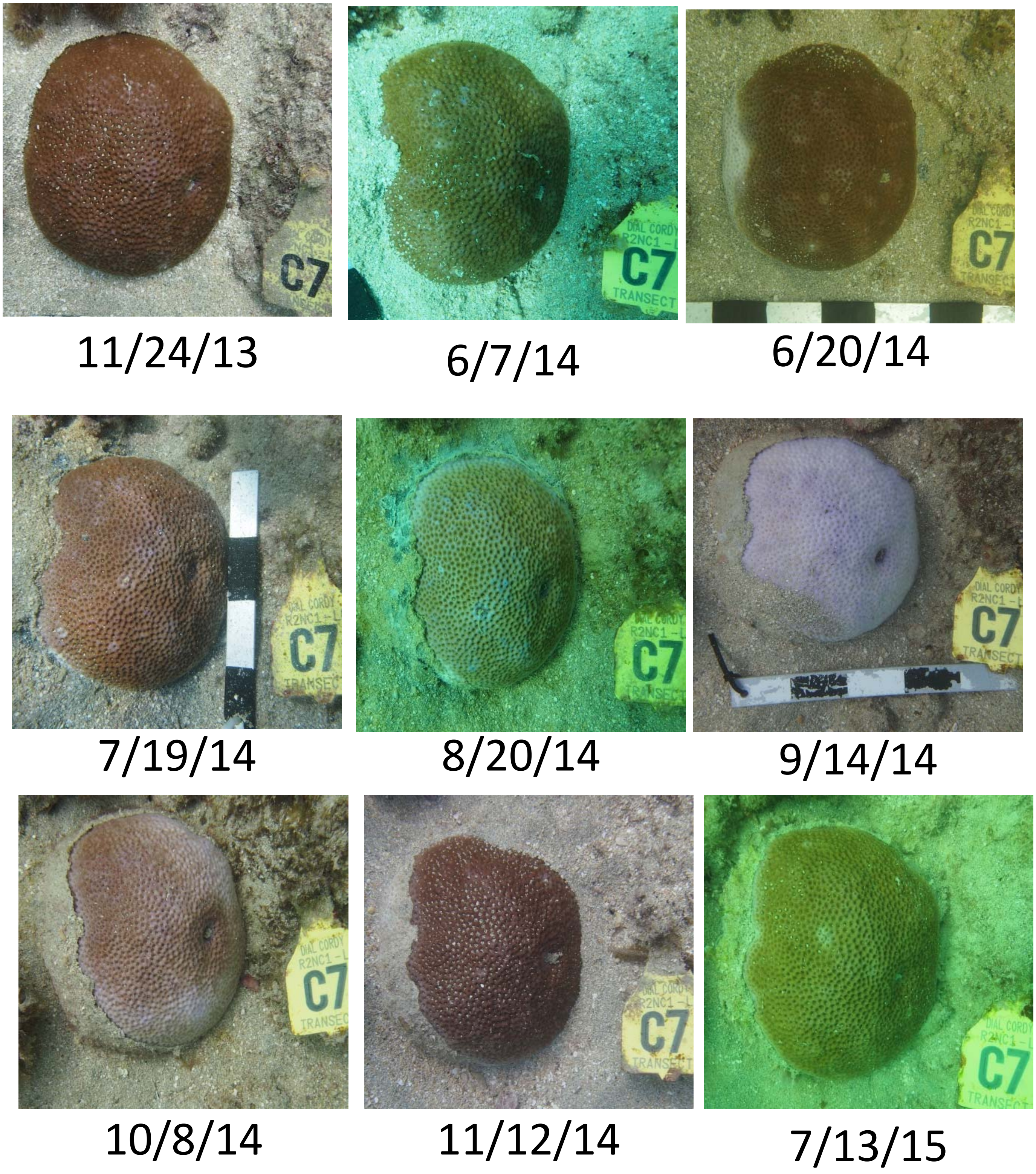

Supplement: Supplemental Figures 1–3 [file peerj-04-2711-s003.pdf]
